# Single Nucleotide Polymorphisms and Dental Fluorosis: A Systematic Review

**DOI:** 10.3390/dj10110211

**Published:** 2022-11-06

**Authors:** Carlos González-Casamada, Martina Nevarez-Rascón, Alfredo Nevarez-Rascón, María González-Galván, Mario Alberto Isiordia-Espinoza, Ronell Bologna-Molina, Leonor Sánchez-Pérez, Nelly Molina-Frechero

**Affiliations:** 1 Health Care Department, Autonomous Metropolitan University Xochimilco, Mexico City 04960, Mexico; 2 Faculty of Dentistry, Autonomous University of Chihuahua, Chihuahua 31000, Mexico; 3 Faculty of Dentistry, National University of Asuncion, Asuncion 1218, Paraguay; 4 Institute of Research in Medical Sciences, Department of Clinics, Los Altos University Center, University of Guadalajara, Tepatitlan de Morelos 47650, Jalisco, Mexico; 5 Research Department, School of Dentistry, Juarez University of the Durango State, Durango 34000, Mexico; 6 Molecular Pathology Area, School of Dentistry, University of the Republic, Montevideo 11200, Uruguay; 7 Division of Biological and Health Sciences, Autonomous Metropolitan University Xochimilco, Mexico City 04960, Mexico

**Keywords:** polymorphism, dental fluorosis, gene

## Abstract

Genetic factors contribute to susceptibility and resistance to fluoride exposure. The aim of this systematic review was to identify alleles/genotypes of single nucleotide polymorphisms (SNPs) associated with dental fluorosis (DF) and to identify them as protective or risk factors. PubMed, ScienceDirect, Cochrane Library, Scopus and Web of Science were searched for articles; the last search was performed in August 2022. Human studies that analyzed the relationship between SNPs and DF published in English were included; systematic reviews and meta-analyses were excluded. Methodological quality was graded using the Joanna Briggs Institute checklist and risk of bias was assessed using the Cochrane Collaboration’s tool. Eighteen articles were included, 44% of which showed high methodological quality and data from 5625 participants aged 6 to 75 years were analyzed. The SNPs COL1A2, ESR2, DLX1, DLX2, AMBN, TUFT1, TFIP11, miRNA17, and SOD2 were considered risk factors, and ESR1, MMP20, and ENAM were considered protective factors. In conclusion, there are alleles and genotypes of different single nucleotide polymorphisms involved in increasing or decreasing the risk of developing dental fluorosis.

## 1. Introduction

At low concentrations, fluoride is considered a compound necessary for human health [1]. It is used in dental preparations to fight tooth decay and is added to water for human consumption in concentrations ranging from 0.5 to 1 mg/L. Exposure to fluoride may vary from region to region, depending on the concentration of fluoride in drinking water and the amount ingested, as well as levels in food and the use of dental preparations. Exposure to fluoride is considerably higher in some areas due to a number of practices [2].

Dental fluorosis (DF) is an undesirable defect in tooth enamel development attributed to an above-optimal systemic exposure to fluoride during critical amelogenesis periods [3]. The severity of the disease depends on when and for how long overexposure occurs, individual response, weight, degree of physical activity, nutritional factors, and bone growth [4], as well as sex and age. Thus, the maxillary central incisor, as a whole, appears most at risk to fluorosis from dietary fluoride between age 15 and 24 months for males and 21 to 30 months for females [5].

Fluoride triggers actions that contribute to the development of DF, including direct effects on ameloblasts and the development and processing of the matrix [6]. By comparing the enamel of animals that consume different concentrations of fluoride in drinking water, it has been shown that the effect of fluoride on ameloblast modulation increased with fluoride dose. There were fewer cycles of cellular modulation with increasing levels of fluoride in drinking water [7]. The pathogenesis of this disease is not entirely clear, and it may be a process involving both genetic factors and environmental factors [8].

Animal studies have determined the involvement of genes in the etiology of DF. It has been shown that genetic factors underlie susceptibility/resistance to dental fluorosis [9,10]. In addition, genes with differences in the pattern of susceptibility to fluorosis in humans living in the same community have been associated with the same environmental exposure. Single nucleotide polymorphisms (SNPs) are useful as genetic markers to identify genes associated with complex diseases. The allele or genotype of a polymorphism will be found more frequently than expected in the case of an association. Depending on the association of polymorphisms, they may act as a protection or risk factor [11].

The identification of SNPs of different genes involved in DF susceptibility and resistance could be a valuable contribution to dental fluorosis prevention strategies. The aim of the present study was to perform a systematic review of current knowledge of the alleles/genotypes of SNPs associated with DF, and to identify them as protective or risk factors.

## 2. Materials and Methods

### 2.1. Protocol and Registration

The protocol was registered in the database of the International Prospective Register of Systematic Reviews (PROSPERO) [12] with the registration number CRD42021297185. This systematic review was performed according to the Preferred Reporting Items for Systematic Reviews and Meta-Analyses (PRISMA) [13] guidelines.

### 2.2. Population, Exposure, Control and Outcome (PECO) Strategy, and Eligibility Criteria

We used the following PECO strategy [14]: P = humans living in the same community, E = involvement of polymorphisms in DF, C = individuals with and without DF or individuals with high and low DF experience, O = associations of polymorphisms with DF.

We included articles that met the following criteria: (1) studies that evaluated the relationship between SNPs and DF; (2) original studies performed on humans; (3) studies in the English language. We excluded case reports, pilot studies, books and/or book chapters, systematic reviews, and meta-analyses.

### 2.3. Information Sources

A digital search was carried out in the PubMed, ScienceDirect, Cochrane Library, Scopus, and Web of Science (WOS) databases. A manual search was performed on the bibliographic references of the selected articles to identify additional studies. The search began in September 2021 and ended in August 2022.

### 2.4. Search Strategy

The keywords that were employed for the advanced search in each database were: dental fluorosis, gene, genetic, skeletal fluorosis, and enamel fluorosis. These words were combined with the Booleans AND NOT as follows: ((dental fluorosis) AND (gene) AND (genetic)), ((gene) AND (dental fluorosis) NOT (skeletal fluorosis)), ((genetic) AND (dental fluorosis) NOT (skeletal fluorosis)), ((enamel fluorosis) AND (gene) AND (genetic)), ((gene) AND (enamel fluorosis) NOT (skeletal fluorosis)), ((genetic) AND (enamel fluorosis) NOT (skeletal fluorosis)). In PubMed, the search was conducted on the titles and abstracts of the articles and the “human” filter was used. In ScienceDirect, Scopus, and Cochrane Library, the search was conducted on the title, abstract, and keywords of the articles. In WOS, the search was conducted on the abstracts of the articles. No publication date restriction was applied in any database, and thus all articles published up to the date of the search were eligible. Articles with restricted access were retrieved through institutional access.

### 2.5. Selection Process

First, duplicated articles were excluded. Then, the titles and abstracts of each article were analyzed and the articles containing relevant information were selected in accordance with the eligibility criteria. Finally, the selected articles were evaluated through full-text analysis in order to determine which of them would be useful for the elaboration of the systematic review. Each reviewer made a list of the articles, which was updated in every step described until they had defined the relationship of the included studies. Two of the reviewers selected the abstracts according to the above criteria, and the concordance of the classification was checked for 20% of the randomly selected publications with a kappa statistic of 0.99 intra-examiner and 0.97 inter-examiner. In the case of a disagreement between the reviewers, a third reviewer was brought in to resolve it.

### 2.6. Data Collection Process

The data required for the systematic review were collected according to standardized forms that contained the most important variables for analysis. Two reviewers performed this process independently, and in the case of a disagreement a third reviewer was brought in to resolve it.

### 2.7. Data Items

The data analyzed for each article were collected in Microsoft Excel^®^ worksheets (2016) in the following order: authors, year of publication of the study, country of the study, study design, range of age of the participants, DF diagnostic index, number of participants, prevalence of DF, extracted samples, laboratory techniques, genes, polymorphisms, alleles, and genotypes.

### 2.8. Methodological Quality Assessment and Risk of Bias

For the evaluation of the methodological quality, the tools of the Joanna Briggs Institute (JBI) [15] were used, and each “yes” response was assigned a point. For cross-sectional studies, scores of 1 to 3 were considered low quality, 4 to 6 moderate, and 7 to 8 high. For case-control studies, scores of 1 to 4 were considered low quality, 5 to 8 moderate, and 9 to 10 high. For cohort studies, scores of 1 to 4 were considered low quality, 5 to 8 moderate, and 9 to 11 high. The Cochrane Collaboration’s [16] tool was employed to evaluate the risk of bias in the selected studies. Each of the five domains was assigned a rating using the terms “low risk”, “some concerns or moderate risk”, and “high risk”. The Risk-Of-Bias VISualization (robvis) [17] tool was used for the elaboration of the risk-of-bias figures. The evaluations of the methodological quality and risk of bias were carried out by two reviewers independently, and in the case of a disagreement a third reviewer participated to resolve it.

## 3. Results

### 3.1. Study Selection

**Figure 1:**
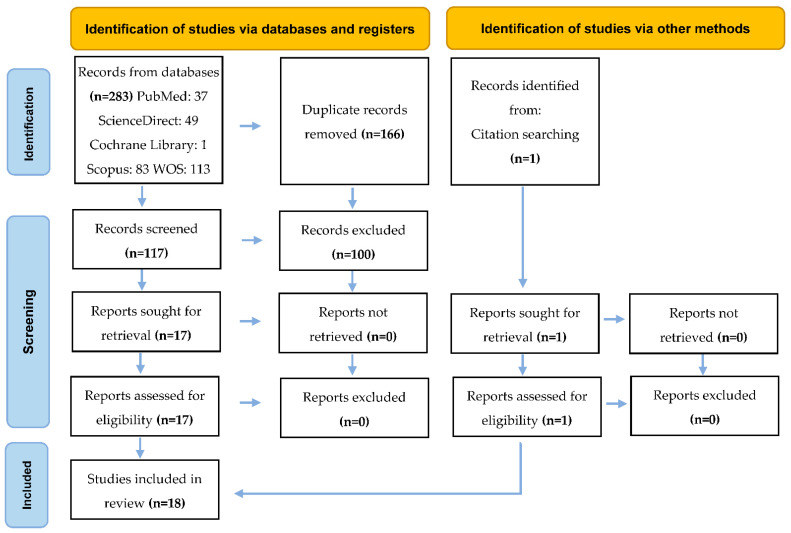
PRISMA 2020 flow diagram for new systematic reviews, which includes searches of databases, registers, and other sources.

Figure 1 presents the article selection process. From the electronic search, a total of 283 articles were registered. First, 166 were excluded because they were duplicates. After reading the titles and abstracts, 100 were excluded because they did not meet the eligibility criteria. The remaining 17 were evaluated in full text and selected for the systematic review. Finally, an article identified among those selected was added.

### 3.2. Study Characteristics

Table 1 shows that the largest number of items came from China (6/18), followed by Brazil (5/18), Mexico (4/18), and India (3/18). Cross-sectional (9/18), case-control (8/18), and cohort (1/18) studies were included. Blood (10/18) and buccal cell (8/18) samples were used for DNA analysis. PCR-RFLP was the most widely used laboratory technique (8/18).

The sample sizes ranged from 30 to 1,017 participants and their age ranged from 6 to 75 years. The Dean method (14/18) and the Thylstrup and Fejerskov index (4/18) were used for the diagnosis of DF. A total of 33% of all participants belonged to the case group and 67% to the control group (Table 2).

**Figure 2:**
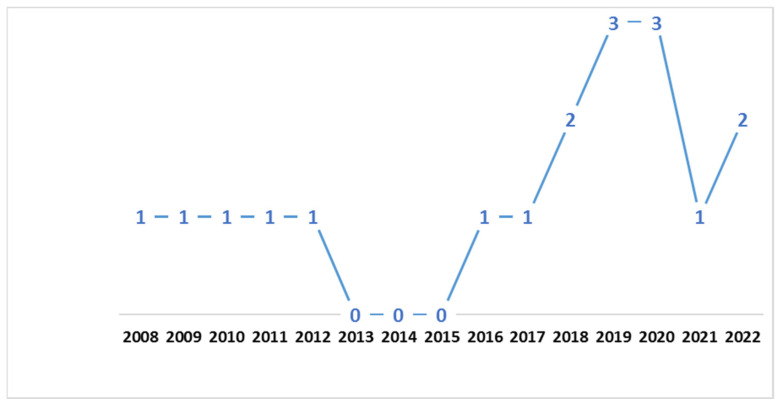
Number of articles selected by year of publication.

Figure 2 shows the number of articles selected per publication year. Most articles (13/18) were published between 2016 and 2022, with the others (5/18) being published between 2008 and 2012. From 2013 to 2015, no articles were published that were included in this systematic review.

### 3.3. Methodological Quality Assessment and Risk of Bias

Table 3 presents the answers to the questions of the methodological quality assessment tools of the JBI. Most of the articles (10/18) were considered to be of moderate quality and the others (8/18) were of high quality.

**Figure 3:**
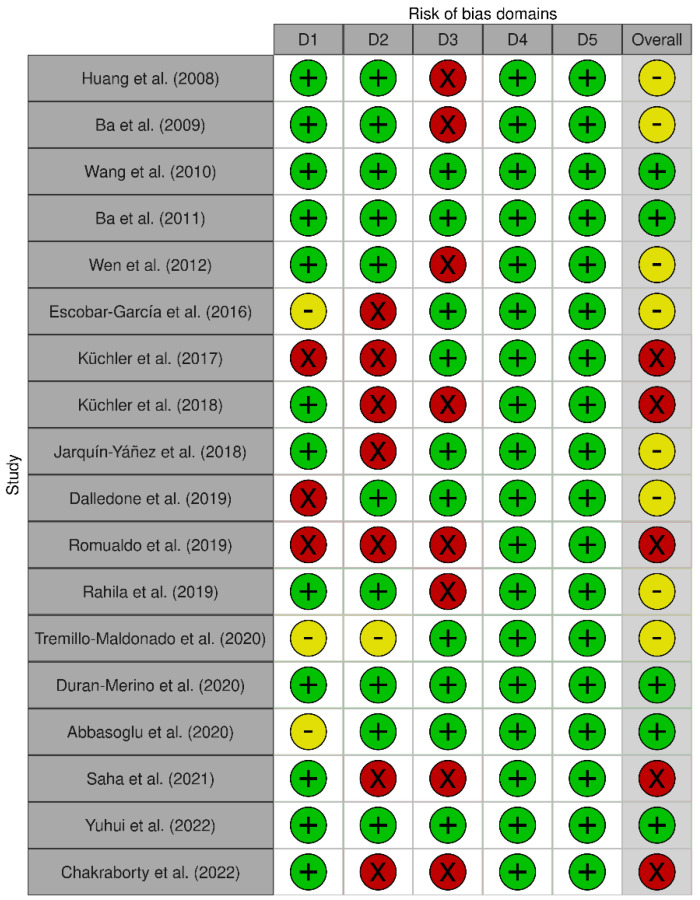
Risk of bias for each selected article. D1: Bias arising from the randomization process. D2: Bias due to deviations from the intended interventions. D3: Bias due to missing outcome data. D4: Bias in measurement of the outcome. D5: Bias in the selection of the report result [18,19,20,21,22,23,24,25,26,27,28,29,30,31,32,33,34,35].

Figure 3 shows the results of the individual evaluation of the risk of bias of the selected articles. Eight articles (44.4%) presented a moderate risk of bias, five articles presented a low risk, and another five presented a high risk (27.8% each).

**Figure 4:**
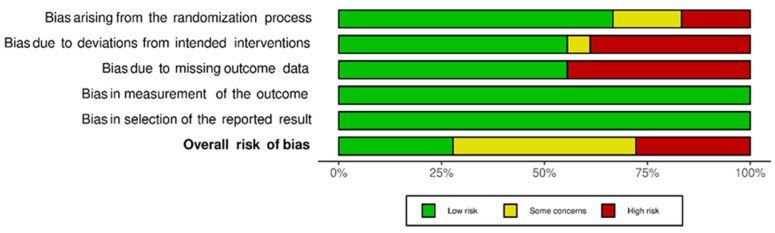
Risk of bias for each domain and risk of general bias.

Figure 4 shows the percentage of risk of bias in each of the domains and the percentage of risk of bias. Despite the results of the risk of bias analysis, the content of the selected articles was relevant to the development of the current systematic review.

### 3.4. Results of Individual Studies

Table 4 and Table 5 expose the alleles and genotypes that were considered risk and protective factors, respectively. Table 6 shows the genotypes that were associated with dental fluorosis but without identifying the type of association.

Table A1, which is annexed in the systematic review, includes information on alleles and genotypes evaluated in the articles included but that were not associated with dental fluorosis.

## 4. Discussion

To our knowledge, this is one of the first systematic reviews to specifically address the relationship between single nucleotide polymorphisms and dental fluorosis. Prior to the extraction and analysis of the data, the seventeen selected articles were evaluated and methodologically qualified. A total of 52 SNPs of 26 candidate genes were studied, of which 18 SNPs of 15 genes were associated with the disease. The SNPs considered risk factors were COL1A2, ESR2, DLX1, DLX2, AMBN, TUFT1, TFIP11, miARN17, and SOD2. On the other hand, those considered protective factors were ESR1, MMP20, and ENAM. In addition, alleles and genotypes of four SNPs were associated without specifying the type of association. No gender differences were mentioned in the results. In comparison with dental caries [36,37] and periodontitis [38], DF has been associated with fewer genes.

Collagen type 1 alpha 2 (COL1A2) is a candidate gene for isolated defects, mainly in dentin [39]. Huang et al. (2008) and Rahila et al. (2019) agreed that the P allele of rs414408 COL1A2 increased the risk of developing dental fluorosis in populations exposed to high levels of fluoride. In addition, Rahila et al. (2019) reported that this polymorphism was associated with the severity of the disease. On the other hand, Jarquín-Yáñez et al. (2018) observed that the C allele of rs412777 increased the risk of the more severe levels of DF, unlike Escobar-García et al. (2016), Saha et al. (2021), and Chakraborty et al. (2022), who did not indicate associations with the same SNP [18,23,26,29,33,35].

Estrogen, which acts through its alpha (ESR1) and beta (ESR2) receptors, promotes the deposition of calcium and phosphorus in bones and plays an important role in the stimulation of osteoblast activity [40,41]. The localization and a pattern of distribution of ER1 immunoreactions in rat ameloblasts throughout the stages of amelogenesis and the involvement of ER1 in epigenetic regulations in pre-ameloblast of the cervical loop of mouse incisors have been demonstrated [42,43,44]. ESR1 rs2234693 was analyzed by four groups with different results. For Ba et al. (2011), the X allele reduced the risk of dental fluorosis in children exposed to high levels of fluoride. In contrast, Dalledone et al. (2019), Saha et al. (2021), and Chakraborty et al. (2022) did not report associations with the same polymorphism. The rs9340799 was evaluated by the same four research groups. The results coincided in the absence of association. Finally, Dalledone et al. (2019) indicated that the C allele of rs12154178 is a protective factor. As for ESR2, for Ba et al. (2011) the R allele of rs1256049 significantly increased the risk in children exposed to high concentrations of fluorides. In contrast, Wang et al. (2010) and Dalledone et al. (2019) agreed on the absence of association. Ending with estrogen receptors, Dalledone et al. (2019) studied six polymorphisms of the Estrogen-Related Receptor Beta (ESRβ) without observing associations with DF [20,21,27,33,35].

Secretory ameloblasts produce the structural proteins amelogenin (AMELX), ameloblastin (AMBN), and enamelin (ENAM) in the enamel matrix [45]. In relation to AMELX rs946252, Küchler et al. (2018) and Tremillo-Maldonado et al. (2020) agreed on the absence of an association [25,30]. Regarding AMBN rs4694075, Küchler et al. (2018) reported that the T allele increased the risk and was associated with moderate and severe phenotypes of dental fluorosis. This coincides with the results of another study, which showed that AMBN gene might serve as the susceptibility factors causing the coal-fired fluorosis in a Chinese population [46]. With respect to ENAM, Duran-Merino et al. (2020) observed that the rs12640848 AG/CT allele was associated with mild phenotypes of the disease in Mexican children, which could be a protective factor; instead, for Küchler et al. (2018), there was no association with the same ENAM polymorphism in Brazilian children. Some considerable differences between these studies are the reported levels of fluoride in drinking water and the severity of dental fluorosis in Mexican children was greater than that presented by Brazilians, in whom the mild phenotype predominated [25,31].

Tuftelin (TUFT1) and members of the microRNA family (miRNA) have already been associated with oral conditions [47,48]. Other studies that showed associations in Brazilian children were those of Küchler et al. (2017) and Abbasoglu et al. (2020). These teams analyzed TUFT1 rs4970957 and miRNA17 rs4284505, indicating that alleles A and G increased the risk of dental fluorosis. In addition, a second team reported that the association occurred with the moderate phenotype of the disease [24,32].

The distal-less (DLX) family are expressed during the early stages of odontogenesis and amelogenesis [49]. Küchler et al. (2017) observed that rs788173 and rs743605 of DLX1 and DLX2, respectively, were associated with the more severe forms of dental fluorosis [24]. It has been indicated that superoxide dismutase (SOD) activity plays an important role in the mechanism of ameloblast apoptosis induced by NaF [50]. Yuhui et al. (2022) evaluated SOD2 rs10370 and rs5746136 and indicated that G and T allele carriers, respectively, had a higher risk of DF [34].

During the secretory stage of amelogenesis, matrix metalloproteinase-20 (MMP20) quickly cleaves proteins that are secreted in the enamel matrix [37]. Romualdo et al. (2019) reported a borderline association between rs1784418 of MMP20 with dental fluorosis. In the same polymorphism, Tremillo-Maldonado et al. (2020) observed an association with the mild phenotypes of the disease, so they considered this SNP a protective factor. Romualdo et al. (2019) observed one polymorphism of MMP2 and another of MMP9 associated with DF without identifying them as protective or risk factors [28,30].

A considerable difference between investigations is the size of the case and control groups. The case groups represented between 22.4 and 27.3% of participants in the studies carried out in Brazil, between 27.4 and 31.25% in China, between 41 and 50% in India, and, lastly, between 66 and 100% in Mexico. Furthermore, comparing the extremes, we noted that in Brazilian cohorts, populations presented more participants with mild than severe DF, constituting the most important subgroup with phenotypes, and they reported lower fluoride concentrations in drinking water than in studies from other countries. On the other hand, Mexican populations presented more moderate/severe phenotypes than mild forms, and reported higher fluoride concentrations in drinking water than in Brazil. Therefore, despite individual heterogeneity, it is suggested that for extreme DF forms the relationship between the concentrations of fluoride in drinking water the ion and the severity of the disease are directly proportional.

Some articles reported incomplete data (sex and age of the participants, concentration of fluoride in water), which could influence the results reported, and prevents us from discussing them in detail. In addition, another limitation of the present study is that there is a high methodological variability (age, number of participants, diagnostic methods, prevalence of DF, origin of the samples, laboratory techniques) between studies included here that makes it difficult to precisely compare the results.

The relevance of the present systematic review lies in strengthening the evidence of the influence of genetic factors on the etiology of dental fluorosis. It is of utmost importance to encourage further research to improve our understanding of the mechanisms of dental fluorosis and thus strengthen prevention strategies for this disease, which covers both oral health and general health.

## 5. Conclusions

Scientific evidence confirms the involvement of single nucleotide polymorphisms in the development of dental fluorosis. Some alleles/genotypes are risk factors, and others are protective factors. Therefore, genetic factors should be considered protagonists in the etiology of this disease, and the continuation of its study is fundamental.

## Figures and Tables

**Table 1:** Characteristics of the included studies.

First Author, Year of Publication	Country	Study Design	Sample	Technique
Huang et al. (2008) [18]	China	Case-control	Blood	PCR-RFLP
Ba et al. (2009) [19]	China	Case-control	Blood	PCR-RFLP
Wang et al. (2010) [20]	China	Case-control	Blood	PCR-RFLP
Ba et al. (2011) [21]	China	Case-control	Blood	PCR-RFLP
Wen et al. (2012) [22]	China	Case-control	Blood	PCR-RFLP
Escobar-García et al. (2016) [23]	Mexico	Cross-sectional	Buccal cells	DNA S
Küchler et al. (2017) [24]	Brazil	Cross-sectional	Buccal cells	RT-PCR
Küchler et al. (2018) [25]	Brazil	Cohort	Buccal cells	RT-PCR
Jarquín-Yáñez et al. (2018) [26]	Mexico	Cross-sectional	Blood	RT-PCR
Dalledone et al. (2019) [27]	Brazil	Cross-sectional	Buccal cells	RT-PCR
Romualdo et al. (2019) [28]	Brazil	Cross-sectional	Buccal cells	RT-PCR
Rahila et al. (2019) [29]	India	Case-control	Blood	PCR-RFLP
Tremillo-Maldonado et al. (2020) [30]	Mexico	Cross-sectional	Buccal cells	DNA S
Duran-Merino et al. (2020) [31]	Mexico	Cross-sectional	Buccal cells	DNA S
Abbasoglu et al. (2020) [32]	Brazil	Cross-sectional	Buccal cells	RT-PCR
Saha et al. (2021) [33]	India	Case-control	Blood	PCR-RFLP
Yuhui et al. (2022) [34]	China	Cross-sectional	Blood	PCR
Chakraborty et al. (2022) [35]	India	Case-control	Blood	PCR–RFLP

PCR-RFLP: Polymerase chain reaction-restriction fragment length polymorphism; DNA S: deoxyribonucleic acid sequencing; RT-PCR: real time-polymerase chain reaction.

**Table 2:** Characteristics of participants and studies.

Reference	Age	Index	*N*	Cases	Controls
Huang et al. (2008) [18]	8–12	Dean	240	75	165
Ba et al. (2009) [19]	8–12	Dean	240	75	165
Wang et al. (2010) [20]	8–12	Dean	237	74	163
Ba et al. (2011) [21]	8–12	Dean	240	75	165
Wen et al. (2012) [22]	8–12	Dean	225	68	157
Escobar-García et al. (2016) [23]	6–12	TF	80	80	0
Küchler et al. (2017) [24]	6–18	Dean	481	108	373
Küchler et al. (2018) [25]	6–18	Dean	1017	255	762
Jarquín-Yáñez et al. (2018) [26]	6–12	TF	230	230	0
Dalledone et al. (2019) [27]	12	Dean	538	147	391
Romualdo et al. (2019) [28]	6–18	Dean	481	108	373
Rahila et al. (2019) [29]	10–30	Dean	120	60	60
Tremillo-Maldonado et al. (2020) [30]	NS	TF	30	26	4
Duran-Merino et al. (2020) [31]	11	TF	71	47	24
Abbasoglu et al. (2020) [32]	10–14	Dean	527	144	383
Saha et al. (2021) [33]	12–75	Dean	87	36	51
Yuhui et al. (2022) [34]	7–13	Dean	649	178	471
Chakraborty et al. (2022) [35]	14–60	Dean	132	71	61

Age in years; NS: not specified; *N*: sample size; TF: Thylstrup and Fejerskov.

**Table 3:** Methodological quality assessment using the JBI checklist.

Articles	Answers											Scores	Quality
	1	2	3	4	5	6	7	8	9	10	11		
Cross-sectional													
Escobar-García et al. (2016) [23]	Y	Y	Y	Y	N	N	Y	Y	--	--	--	6	Moderate
Küchler et al. (2017) [24]	U	U	Y	Y	Y	Y	Y	Y	--	--	--	6	Moderate
Jarquín-Yáñez et al. (2018) [26]	Y	Y	Y	Y	N	N	Y	Y	--	--	--	6	Moderate
Dalledone et al. (2019) [27]	Y	Y	Y	Y	Y	U	Y	Y	--	--	--	7	High
Romualdo et al. (2019) [28]	U	Y	Y	Y	Y	Y	Y	Y	--	--	--	7	High
Tremillo-Maldonado et al. (2020) [30]	Y	N	Y	Y	N	N	Y	Y	--	--	--	5	Moderate
Duran-Merino et al. (2020) [31]	Y	N	Y	Y	N	N	Y	Y	--	--	--	5	Moderate
Abbasoglu et al. (2020) [32]	Y	Y	Y	Y	N	N	Y	Y	--	--	--	6	Moderate
Yuhui et al. (2022) [34]	Y	Y	N	Y	Y	Y	Y	Y	--	--	--	7	High
Case-control													
Huang et al. (2008) [18]	Y	Y	Y	N	Y	Y	Y	Y	Y	Y	--	9	High
Ba et al. (2009) [19]	Y	Y	Y	N	Y	Y	Y	Y	Y	Y	--	9	High
Wang et al. (2010) [20]	Y	Y	Y	U	Y	Y	Y	Y	Y	Y	--	9	High
Ba et al. (2011) [21]	Y	Y	Y	Y	Y	Y	Y	Y	Y	Y	--	10	High
Wen et al. (2012) [22]	Y	Y	Y	N	Y	Y	Y	Y	Y	Y	--	9	High
Rahila et al. (2019) [29]	Y	Y	Y	N	Y	Y	U	Y	Y	Y	--	8	Moderate
Saha et al. (2022) [33]	Y	Y	Y	N	Y	Y	U	N	Y	Y	--	7	Moderate
Chakraborty et al. (2022) [35]	Y	Y	Y	N	Y	Y	U	N	Y	Y	--	7	Moderate
Cohort													
Küchler et al. (2018) [25]	U	Y	Y	Y	Y	Y	Y	N	N	N	Y	7	Moderate

Y: yes, N: no, U: unclear.

**Table 4:** Alleles and genotypes considered risk factors.

Author	Gene: SNP	Results
Huang et al. (2008) [18]	COL1A2: rs414408	P allele increased the risk of DF (OR = 4.85, IC95%: 1.22–19.32).
Ba et al. (2011) [21]	ESR2: rs1256049	R allele increased the risk of DF (OR = 1.821; IC95%: 1.013–3.274).
Küchler et al. (2017) [24]	DLX1: rs788173 DLX2: rs743605	In both SNPs, AG genotypes were associated with the most severe form of DF (*p* < 0.05).
Küchler et al. (2018) [25]	AMBN: rs4694075	T allele increased the risk of DF (*p* < 0.0001; OR = 2.12, 95% CI: 1.51–2.97)
	TUFT1: rs4970957	A allele increased the risk of DF (*p* < 0.049; OR = 1.42, 95% CI: 1.00–2.02).
	TFIP11: rs5997096	T allele was associated with the moderate/severe form of DF (*p* = 0.005).
Jarquín-Yáñez et al. (2018) [26]	COL1A2: rs412777	C allele increased the risk of DF (*p* = 0.05, OR = 2.59, IC95%: 1.60–4.20)
Rahila et al. (2019) [29]	COL1A2: rs414408	PP and Pp genotypes increased the risk of DF (OR = 31.4; IC95%: 3.9–48.7 and OR = 4.0, IC95%: 1.6–10.1)
Abbasoglu et al. (2020) [31]	miARN17:rs4284505	G allele increased the risk of DF (*p* = 0.031; OR = 2.26, IC95%: 1.04–4.73)
Yuhui et al. (2022) [34]	SOD2: rs10370	G allele increased the risk of DF (IC95% for OR: 1.20, 2.96)
	SOD2: rs5746136	T allele increased the risk of DF (IC95% for OR: 1.09, 2.69)

**Table 5:** Alleles and genotypes considered protective factors.

Author	Gene: SNP	Results
Ba et al. (2011) [21]	ESR1: rs2234693	X allele decreased the risk of DF (OR = 0.542, IC95%: 0.314–0.936).
Dalledone et al. (2019) [27]	ESR1: rs12154178	CC genotype decreased the risk of DF (*p* = 0.038; OR = 0.51, IC95%: 0.7–0.97).
Tremillo-Maldonado et al. (2020) [30]	MMP20: rs1784418	TG allele variant decreased the risk of DF (*p* = 0.001).
Duran-Merino et al. (2020) [31]	ENAM: rs12640848	AG/CT allele variant was associated with a lower severity of DF (*p* = 0.000).

**Table 6:** Associated genotypes without identifying their function.

Author	Gene: SNP	Results
Küchler et al. (2017) [24]	TIMP1: rs4898	Borderline association with DF was observed in CT genotype (*p* = 0.073).
Romualdo et al. (2019) [28]		Borderline association with DF was observed in:
	MMP2: rs243865	CT genotype (*p* = 0.06).
	MMP9: rs17576	AG genotype (*p* = 0.08).
	MMP20: rs1784418	AG genotype (*p*= 0.06).

## Data Availability

Datasets generated during and/or analyzed during the current study are available from the corresponding author upon reasonable request.

## Appendix A

**Table A1:** Alleles and genotypes not associated with DF.

Authors	Gene: SNP		Allele/Genotype
Huang et al. (2008) [18]	COL1A2:	rs4266	rr/Rr/RR
Ba et al. (2009) [19]	OC:	rs1800247	hh/Hh/HH
Wang et al. (2010) [20]	ESR2:	RsaI	rr/Rr/RR
Ba et al. (2011) [21]	ESR1:	rs9340799	pp/Pp/PP
Wen et al. (2012) [22]	PTH:	Bst BI	bb/Bb/BB
Escobar-García et al. (2016) [23]	COL1A2:	rs412777	A/C
Küchler et al. (2017) [24]	TIMP2:	rs7501477	G/T
	MMP13:	rs2252070	A/G
Küchler et al. (2018) [25]	ENAM:	rs12640848	A/G
	AMELX:	rs946252	A/G
Dalledone et al. (2019) [27]	ESR1:	rs1884051	A/G
		rs9340799	A/G
		rs2234693	C/T
	ESR2:	rs4986938	A/G
		rs1256049	A/G
	ESRRβ:	rs745011	C/T
		rs6574293	A/G
		rs10132091	C/T
		rs4903399	C/T
		rs1077430	A/G
		rs1676303	C/T
Romualdo et al. (2019) [28]	MMP20:	rs1711437	A/G
	MMP2:	rs243867	A/G
Tremillo-Maldonado et al. (2020) [30]	AMELX:	rs946252	C/T
	ODAM:	rs1514392	G
Saha et al. (2021) [33]	ESR1:	rs2234693	T/C
		rs2228480	G/A
		rs3798577	T/C
		rs2077647	T/C
		rs9340799	A/G
		rs2077647	T/C
	COL1A2:	rs42524	C/G
		rs412777	A/C
	BGLAP:	rs1800247	T/C
	SPARC:	rs6579885	G/C
		rs4958278	T/C
Yuhui et al. (2022) [34]	SOD2:	rs4880	A/G
	SOD3:	rs13306703	C/T
		rs2855262	T/C
Chakraborty et al. (2022) [35]	ESR1:	rs2077647	C/T
		rs2234693	C/T
		rs9340799	A/G
		rs2228480	A/G
		rs3798577	C/T
	COL1A2:	rs412777	A/C
		rs42524	C/G
	BGLAP:	rs1543294	C/T
		rs1800247	C/T
		rs759330	A/G
	SPARC:	rs4958278	C/T
		rs1800247	C/G
		rs6579885	C/T
	VDR:	rs731236	A/G
	MMP20:	rs2285053	C/T
